# Molecular Cloning, Characterization, and mRNA Expression of Hemocyanin Subunit in Oriental River Prawn* Macrobrachium nipponense*

**DOI:** 10.1155/2016/6404817

**Published:** 2016-10-13

**Authors:** Youqin Kong, Liqiao Chen, Zhili Ding, Jianguang Qin, Shengming Sun, Ligai Wang, Jinyun Ye

**Affiliations:** ^1^School of Life Sciences, East China Normal University, Shanghai 200062, China; ^2^School of Life Sciences, Huzhou University, Huzhou, Zhejiang 313000, China; ^3^School of Biological Sciences, Flinders University, Adelaide, SA 5001, Australia

## Abstract

Hemocyanin is a copper-containing protein with immune function against disease. In this study, a hemocyanin subunit named MnHc-1 was cloned from* Macrobrachium nipponense*. The full-length cDNA of MnHc-1 was 2,163 bp with a 2,028-bp open reading frame (ORF) encoding a polypeptide of 675 amino acids. The MnHc-1 mRNA was expressed in the hepatopancreas, gill, hemocytes, intestine, ovary, and stomach, with the highest level in the hepatopancreas. In the infection trial, the MnHc-1 mRNA transcripts in the hemocytes were significantly downregulated at 3 h after injection of* Aeromonas hydrophila* and then upregulated at 6 h and 12 h, followed by a gradual recovery from 24 to 48 h. The MnHc-1 transcriptional expression in the hepatopancreas was measured after* M. nipponense* were fed seven diets with 2.8, 12.2, 20.9, 29.8, 43.1, 78.9, and 157.1 mg Cu kg^−1^ for 8 weeks, respectively. The level of MnHc-1 mRNA was significantly higher in the prawns fed 43.1–157.1 mg Cu kg^−1^ diet than in that fed 2.8–29.8 mg Cu kg^−1^ diet. This study indicated that the MnHc-1 expression can be affected by dietary copper and the hemocyanin may potentially participate in the antibacterial defense of* M. nipponense*.

## 1. Introduction

Crustaceans like other invertebrates only have innate immunity, including many immune molecules to eliminate exogenous pathogens [1]. To date, many studies have verified that hemocyanin is an important nonspecific innate immune defense molecule and can provide an effective immune defense in arthropods [2–8]. Hemocyanin is a copper-containing multifunctional protein in mollusks and arthropods [9]. Its primary function is to transport and store oxygen and also to participate in osmoregulation, molt cycle, exoskeleton formation, and melanin synthesis [10–12]. So far, studies on the immunologic function in crustacean are mainly focused on the hemocyanin itself or its degraded peptides, which have hemolytic activity, agglutination property, and antiviral function [2, 4, 5, 7, 13]. Hemocyanin genes have been cloned and characterized in certain crustacean species such as* Litopenaeus vannamei* [14],* Eriocheir sinensis* [15],* Cherax quadricarinatus* [16],* Homarus americanus* [17], and* Caridina multidentata *and* Atyopsis moluccensis* [18].

Copper is a central component of hemocyanin and a cofactor of many other enzymes, like superoxide dismutase, cytochrome oxidase, and lysyl oxidase [19, 20]. The dietary copper level can affect crustacean immune responses in* Penaeus monodon* [21] and* E. sinensis* [22]. It was found that the expression of hemocyanin mRNA in* E. sinensis* was affected by dietary copper level [15]. Investigation of the functional relationship between dietary copper and hemocyanin mRNA expressions can provide better understanding on crustacean innate immunity and offer insight into disease control through dietary management in shrimp farming. We hypothesize that copper as a major component of hemocyanin can impact prawn innate immunity through dietary copper manipulation.

Oriental river prawn (*Macrobrachium nipponense*) is an important aquaculture species in China and other Southeast Asian countries [23]. Various diseases have been found in the* M. nipponense* farming population due to intensive culture and environmental pollution [24]. For example, the bacterial disease induced by* Aeromonas hydrophila* is one of the major diseases which can cause 30% death of prawn, sometimes as high as 70% [25]. As a result, the investigation on the* M. nipponense* innate immune mechanism against* A. hydrophila* has become a key issue of health management in crustacean farming. The hemocyanin subunit is a functional group for crustacean immunity, and a hemocyanin subunit has been cloned and characterized in the freshwater prawn* M. nipponense* [26]. In this study, we discovered another hemocyanin subunit in* M. nipponense *(MnHc-1). To understand the role of MnHc-1 in* M. nipponense *immunization, (1) the full-length cDNAs of MnHc-1 was cloned, (2) the distribution of MnHc-1 in different tissues was examined, (3) the mRNA expression of MnHc-1 in immune defense after prawn challenge with* A. hydrophila* was examined, and (4) the relative expression level of MnHc-1 in the hepatopancreas of* M. nipponense* fed different levels of copper was quantified.

## 2. Materials and Methods

### 2.1. Sample Collection and Infection Test

All adult oriental river prawns (*M. nipponense*) were obtained from a local farm in Shanghai. The hepatopancreas, muscle, gill, ovary, intestine, heart, and stomach were collected from healthy prawn, flash-frozen in liquid nitrogen, and stored at −80°C until RNA extraction. A total of 1 mL hemolymph was collected from the ventral sinus using a sterile syringe and diluted using half volume of anticoagulant solution [27], then centrifuged at 8000 ×g for 10 min at 4°C to collect the hemocyte, and stored at −80°C immediately until RNA extraction.

Prior to the challenge experiment, the adult prawns were acclimatized in the laboratory for 2 weeks. A total of 300 healthy prawns were randomly divided into two groups with five replicates. According to the preliminary experiment, the prawn in the bacterial challenge trial was injected with 100 *μ*L* A. hydrophila* in saline suspension (1 × 10^7^ CFU/mL) obtained from Shanghai Ocean University, while each prawn in the control group received the same volume of saline injection. After injection, prawns were put back to the rearing tanks, and hemocyte samples were collected at 0, 3, 6, 12, 24, and 48 h after injection, centrifuged, and stored at −80°C for RNA extraction.

### 2.2. Experimental Diets and Farming

The juvenile* M. nipponense* were obtained from the same farm as the adults and acclimated for two weeks in the laboratory conditions prior to the feeding trial. The basal diet was supplemented with copper sulphate (Analytical Reagent, Shanghai Chemical Co., Shanghai, China) at 0, 10, 20, 30, 40, 80, and 160 mg Cu kg^−1^ diet, respectively. Procedure of diet preparation was similar to that described by Li et al. [28], and all other required nutrients for* M. nipponense* were included. The actual copper concentrations in the feeds were analyzed to be 2.8, 12.2, 20.9, 29.8, 43.1, 78.9, and 157.1 mg kg^−1^, respectively, by the flame atomic absorption photometry [29]. The compositions of the experimental diet were showed in Table 1.

Prawn juveniles (0.101 ± 0.001 g) were randomly placed in 21 of 300-L tanks with 30 prawns per tank in triplicate. Prawns were fed to apparent satiation twice daily (8:00 and 17:00 h) for 56 days. To maintain water quality, one-third of the tank water was exchanged daily. During the feeding period, water temperature was 27–30°C, dissolved oxygen >6.5 mg L^−1^, and total ammonia nitrogen <0.1 mg L^−1^. The Cu concentration in rearing water was 1.4–1.7 *μ*g Cu L^−1^. At the end of the feeding trial, all prawns were counted and gained survival rate (Survival rate = 100 × (final prawn number)/(initial prawn number)). Prawns were fasted for 24 h before the hepatopancreas was collected. These samples were stored at −80°C until RNA extraction.

### 2.3. RNA Extraction and Reverse Transcription

Total RNA was isolated using RNA extraction kit (Aidlab Biotech, Beijing, China) following manufacture protocol. In reverse transcription reaction, 3 *μ*g of total RNA was used for synthesis of first-strand cDNA by the PrimeScript™ RT-PCR Kit (TaKaRa, Dalian, China). Acquired cDNA was stored at −20°C for subsequent quantitative real-time PCR (qRT-PCR).

### 2.4. 3′-RACE and 5′-RACE Amplification of MnHc-1 Gene, Cloning, and Sequencing

To get the full length of MnHc-1 cDNA sequence, SMART™ RACE cDNA Amplification Kit (Clontech, USA) was used to conduct the 3′-RACE and 5′-RACE. Gene-specific primers of MnHc-1 were designed based on the known fragments initially identified from the EST cDNA library of* M. nipponense* [30]. The total RNA of the mixed hepatopancreas was used as a template. PCR was performed in a Bio-Rad thermal cycler. Reaction volume was 50 *μ*L containing 5.0 *μ*L 10x Ex Taq Buffer (Mg^2+^ Plus), 5.0 *μ*L 10x UPM, 4.0 *μ*L cDNA template, 4.0 *μ*L dNTPs mix (2.5 mM each), 1.0 *μ*L GSP (10 *μ*M), 0.26 *μ*L Ex Taq HS (TaKaRa, Dalian, China), and 30.74 *μ*L of sterile deionized water. The PCR conditions were set as follows: 5 cycles of 94°C for 30 s and 72°C for 3 min, then 5 cycles of 94°C for 30 s and 70°C for 30 s, and 25 cycles of 94°C for 30 s, 68°C for 30 s, and 72°C for 3 min. All used primers were summarized in Table 2. Primers of MnHc-s1 and MnHc-a1 were used to confirm the MnHc-1 fragment from the EST cDNA library. 5′-CDS primer A (as the RT primer), gene-specific primer of MnHc-5′GSP, and the UPM (universal primer A mix) were used for the 5′-RACE. 3′-CDS primer A (as the RT primer), gene-specific primer of MnHc-3′GSP, and the UPM were used for the 3′-RACE.

The PCR products were purified by the UNIQ-10 Gel Extraction Kit (Sangon, Shanghai, China) and cloned into the pUCm−T vector (Sangon, Shanghai, China). The transformed bacteria were identified, confirmed by blue/white screening, and validated by PCR. More than two recombinant plasmids were sequenced using BigDye Terminator v3.1 Cycle Sequencing Kit operating on an Automatic DNA Sequencer (ABI 3730xl DNA Analyzer).

### 2.5. Sequence Analysis

The online ORF finder program (http://www.ncbi.nlm.nih.gov/gorf/) was used to predict the gene's putative open reading frame. The BLAST algorithm at the National Center for Biotechnology Information (http://www.ncbi.nlm.nih.gov/BLAST/) was applied to search for sequence homology. SMART (Simple Modular Architecture Research Tool, http://smart.embl-heidelberg.de/) was used to predict conserved motifs. Signal sequence was carried out using SignalP 4.1 program (http://www.cbs.dtu.dk/services/SignalP/). The deduced amino acid sequence of MnHc-1 from* M. nipponense* and other invertebrate hemocyanin sequences acquired from NCBI database were aligned by software ClustalX. A neighbor-joining (NJ) phylogenetic relationship was established based on amino acid sequences of the hemocyanin using the MEGA 5.1 program (http://www.megasoftware.net/).

### 2.6. Analysis of the MnHc-1 Expression in Tissues

The mRNA expression of MnHc-1 in the hepatopancreas, muscle, gill, ovary, intestine, heart, and stomach was detected by qRT-PCR with *β*-actin as internal control. The synthesis of the first-strand cDNA was the same as that described above. Gene-specific primers of MnHc-s2, MnHc-a2, *β*-actin-s, and *β*-actin-a were used in qRT-PCR (Table 2). The qRT-PCR liquid compositions and conditions were according to the manufacturer instructions of SYBR Premix Ex Taq (TaKaRa, Dalian, China). The qRT-PCR was conducted on the CFX96™ Real-Time System (Bio-Rad, USA) according to the manufacturer's protocols. During the detection, each sample was run in triplicate. The melt curve of the amplification products was analyzed to ensure that only one PCR product was amplified and detected at the end of each PCR. Expression level of MnHc-1 was measured by 2^−ΔΔCt^ method [31].

### 2.7. Analysis of the MnHc-1 Expression after* A. hydrophila* Challenge

The mRNA expression of MnHc-1 in the hemocytes of* M. nipponense* injected with* A. hydrophila* or saline water was, respectively, detected at 0, 3, 6, 12, 24, and 48 h after injection by a quantitative real-time RT-PCR.

### 2.8. Analysis of the MnHc-1 Expression after the Feeding Treatment at Different Dietary Copper Levels

At the feeding experiment, mRNA expression of MnHc-1 in hepatopancreas of* M. nipponense* from the feeding treatment at seven copper levels was analyzed by qRT-PCR.

### 2.9. Statistical Analysis

All the data are expressed as means ± SD. SPSS software (version 16.0) was used for statistical analysis. The results of relative mRNA expression in challenge test were analyzed by *t*-test, while other results were subjected to one-way ANOVA and post hoc Duncan multiple range tests. Differences were regarded as significant at *P* < 0.05.

## 3. Results

### 3.1. Sequence Analysis of MnHc-1

**Figure 1:**
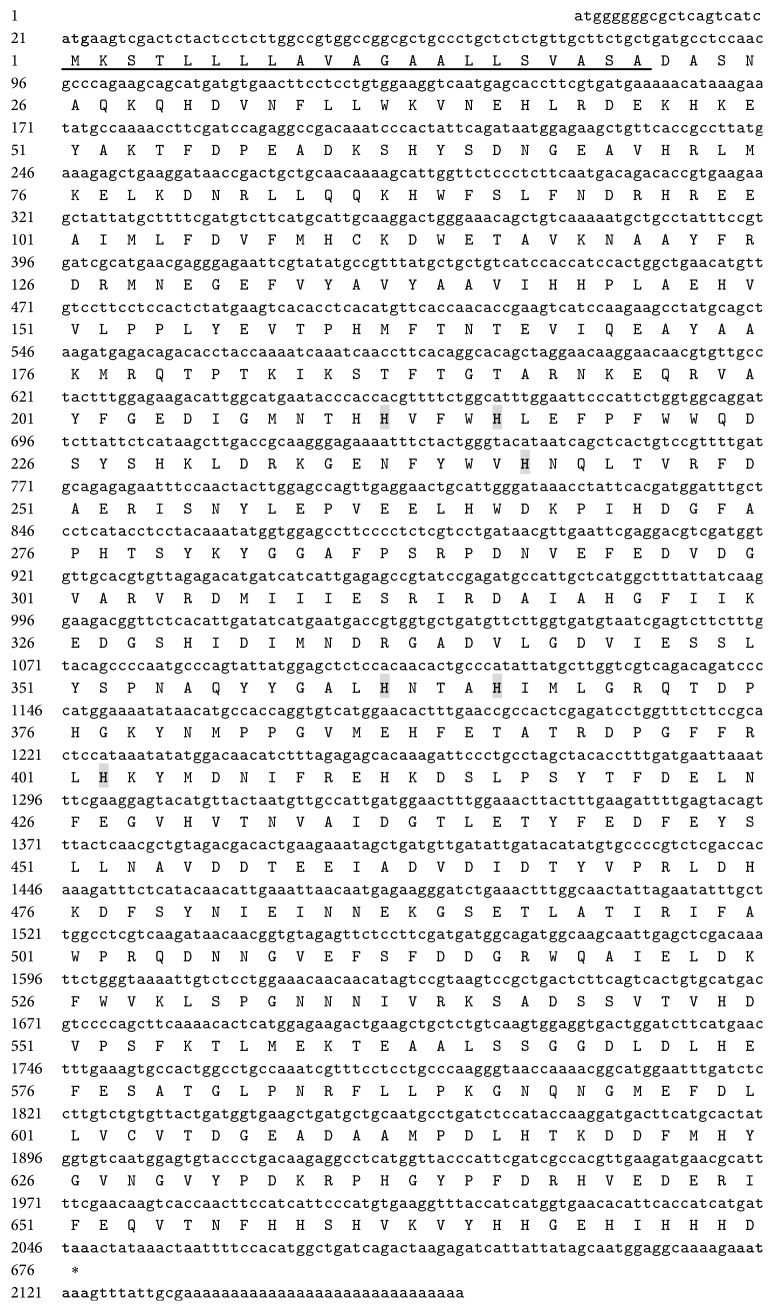
*M. nipponense* hemocyanin subunit 1 cDNA and deduced amino acid sequences. Putative signal peptide sequences were underlined. The start codon (atg), stop codon (taa), and the polyadenylation signal sequence (aataaa) were marked in bold and the six histidine residues within the copper-binding sites were marked in bold and shadow background.

The full length of MnHc-1 cDNA from the* M. nipponense* was 2,163 bp (GenBank accession number JX456149.1), containing a 20 bp 5′-untranslated region, a 115 bp 3′-untranslated region with poly A tail, and a 2,028 bp open reading frame (ORF) (Figure 1). The ORF encoded a polypeptide of 675 amino acids with a calculated molecular weight of 78.060 kDa and an isoelectric point of 5.45. A putative signal peptide of 21 amino acids was found in the N-terminus by using SignalP 4.1 program [32]. SMART program predicted that the MnHc-1 belongs to the hemocyanin family, including N terminal domain (Ser24-Val150), typical copper-containing domain (Pro154-Glu411), and C terminal domain (Pro417-His667 amino acid). Six histidine residues (H212, H216, H242, H362, H366, and H402) of copper-binding sites were identified [17].

### 3.2. Homology and Phylogenetic Analysis of MnHc-1

**Figure 2:**
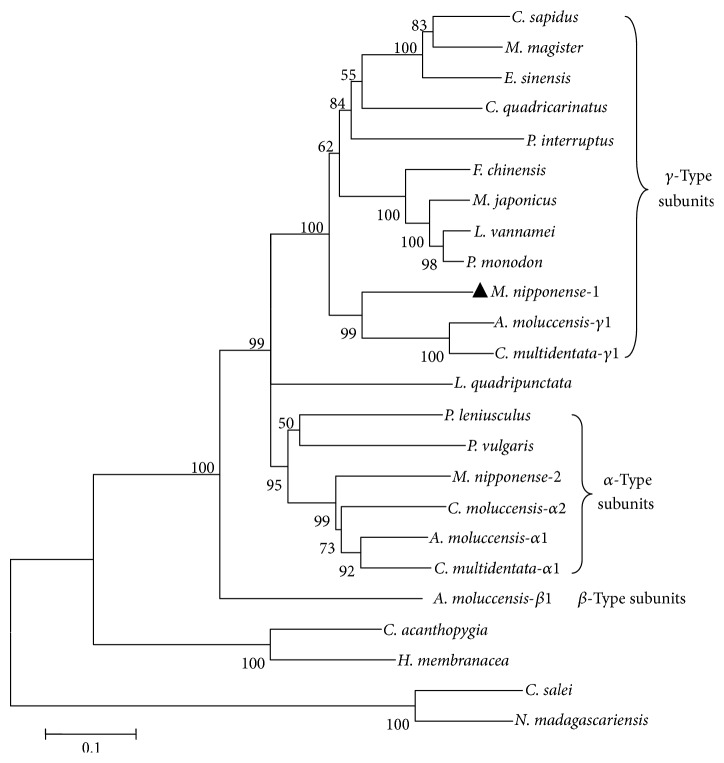
The phylogenetic tree based on the sequences of hemocyanin from different species. The amino acid sequences were derived from the GenBank as the following accession numbers:* A. moluccensis*-*α*1 (CCF55379.1);* A. moluccensis*-*β*1 (CCF55382.1);* A. moluccensis*-*γ*1 (CCF55383.1);* C. acanthopygia* (CAR85694.1);* C. multidentata-α*1 (CCF55384.1);* C. multidentata-α*2 (CCF55385.1);* C. multidentata*-*γ*1 (CCF55387.1);* C. quadricarinatus* (AFP23115.1);* C. salei* (CAC44753.1);* C. sapidus *(AAF64305.1);* E. sinensis* (AEG64817.1);* F. chinensis* (ACM61982.1);* H. membranacea* (CAR85695.1);* L. quadripunctata* (ADE58571.1);* L. vannamei* (ADZ15149.1);* M. japonicus* (ABR14693.1);* M. magister* (AAW57893.1);* M. nipponense*-1 (AGA17871.1);* M. nipponense*-2 (AEC46861.1);* N. madagascariensis* (CAD68057.1);* P. interruptus* (AAB22190.1);* P. leniusculus* (AAO47336.1);* P. monodon *(AEB77775.1); and* P. vulgaris* (CAC69244.1).

Homology analysis with BLAST algorithm showed that MnHc-1 amino acid sequences had 75%, 73%, 70%, and 68% similarity to that of the* A. moluccensis* gamma subunit,* P. monodon*,* C. quadricarinatus,* and* E. sinensis*, respectively. The MnHc-1 exhibited 64% identity with another subunit of hemocyanin in* M. nipponense *(MnHc-2, GenBank accession number JF683437.1). A phylogenetic tree was constructed with the 24 full-length hemocyanin sequences from arthropod based on the neighbor-joining method (Figure 2). The result of phylogenetic analysis revealed that MnHc-1 was more closely related to the hemocyanin gamma subunit 1 of freshwater shrimps* A. moluccensis* and* C. multidentata*.

### 3.3. Analysis of MnHc-1 mRNA Expressions in Tissues

**Figure 3:**
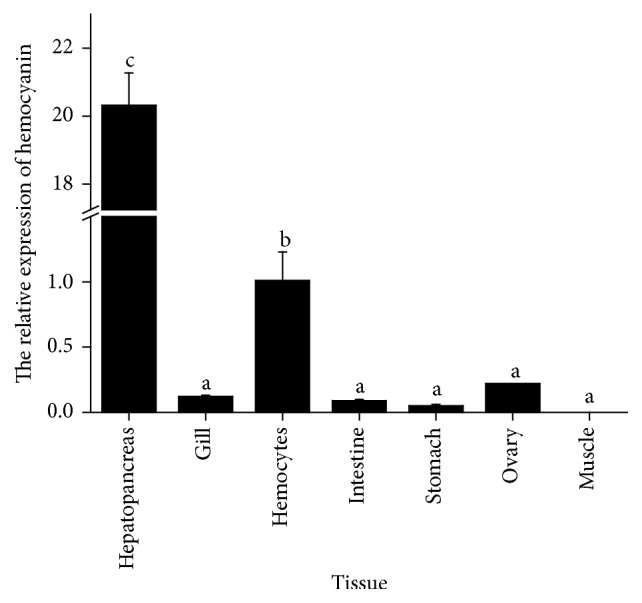
Quantitative real-time PCR analysis of hemocyanin expressions in various tissues of* M. nipponense*. The *β*-actin gene was used as the internal control. Different letters in each index indicated significant differences (*P* < 0.05).

The mRNA transcripts of MnHc-1 were analyzed in the tissues of hepatopancreas, gill, muscle, hemocytes, intestine, ovary, and stomach. In prawn, the highest expression was found in hepatopancreas (*P* < 0.05); the expression value is 20.32, which is 20 times higher or more compared with other tissues. The value is as low as 0 so that the gene is not expressed in muscle (Figure 3). It looks to us that its expression is tissue-specific.

### 3.4. Analysis of MnHc-1 mRNA Expression after Challenge with* A. hydrophila*

**Figure 4:**
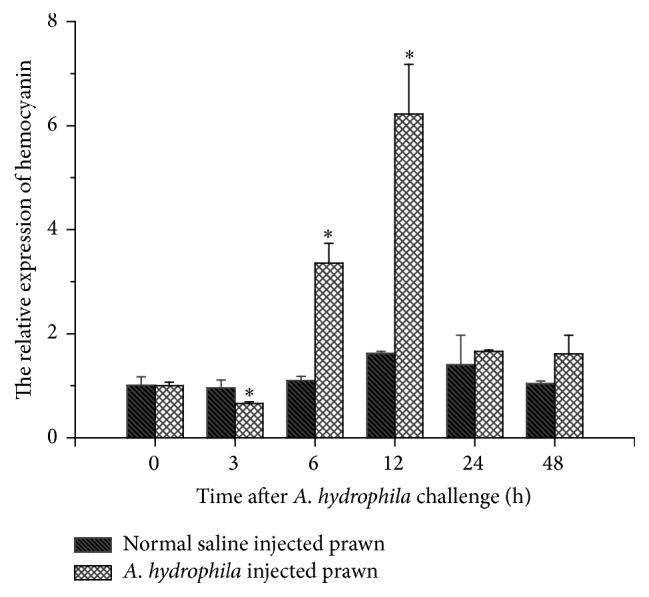
The hemocyanin gene expression in hemocytes of* M. nipponense*. The statistical test was performed by *t*-test after challenge with* A. hydrophila* compared to the control at the same time points (0, 3, 6, 12, 24, and 48 h). The internal standard was *β*-actin gene. Asterisks indicate being significantly different (*P* < 0.05).

Figure 4 exhibits the expression profile of MnHc-1 in the hemocytes challenged by* A. hydrophila* from 0 to 48 h. The expression of MnHc-1 showed a distinct time-dependent pattern. The mRNA expression level significantly dropped at 3 h after injection (0.6 times) (*P* < 0.05) and then started to significantly increase and reached the peak in 12 h (6.2 times) (*P* < 0.05), followed by a recovery to the initial level in 24 h and 48 h.

### 3.5. Analysis of MnHc-1 mRNA Expression in Response to Graded Levels of Dietary Copper

**Figure 5:**
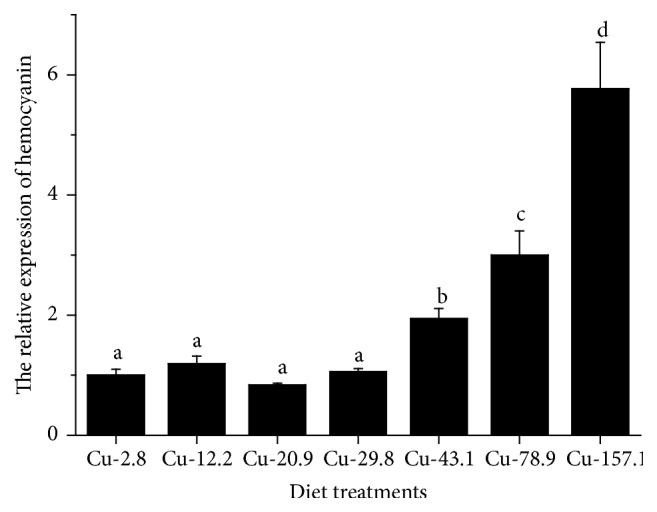
Relative hemocyanin mRNA levels in hepatopancreas of* M. nipponense. M. nipponense* was fed diet with different levels of copper for 8 weeks. Hemocyanin mRNA levels were evaluated by qRT-PCR and expressed relatively to the level of *β*-actin mRNA. Different letters in each index indicated significant differences (*P* < 0.05).

Survival rate (70–81%) of prawns was not affected by the dietary copper levels. As shown in Figure 5, the response of MnHc-1 expression in the hepatopancreas was affected by the dietary copper level. With the dietary copper level increasing from 43.1 to 157.1 mg Cu kg^−1^ diet, the level of MnHc-1 mRNA in hepatopancreas of prawns significantly increased (1.9-fold to 5.8-fold) (*P* < 0.05) and was significantly higher compared to that fed 2.8–29.8 mg Cu kg^−1^ diet (*P* < 0.05), but differences in 2.8–29.8 groups were not significant (*P* > 0.05).

## 4. Discussion

Hemocyanin is an extracellular, multisubunit protein in crustacean [14, 33]. Those subunits differ considerably in their primary structures and are encoded by distinct genes [34]. In this study, we cloned and characterized the expression pattern of one hemocyanin subunit from* M. nipponense *(MnHc-1). MnHc-1 was a polypeptide of 675 amino acids with a 21-amino acid putative signal peptide. The signal peptide ends Ala-X-Ala motif, which is a frequent accordance prior to the cleavage site of signal peptides, suggesting that a cleavage site is located at the 21-22 amino acids [32, 35]. Structurally, MnHc-1 has conservative copper-binding domains including six histidine residues (H212, H216, H242, H362, H366, and H402) of the copper-binding sites; this domain agrees with other crustaceans [4, 14, 15]. Based on immunological methods, the crustacean hemocyanin subunits are classified into three distinct subunit types: alpha, beta, and gamma [9]. The phylogenetic analysis showed that MnHc-1 and MnHc-2 belong to separate clade; MnHc-1 belongs to the gamma subunit which has evolved at a later time compared to alpha and beta subunits in freshwater shrimps [18].

The present study showed that the highest level of MnHc-1 mRNA expression occurred in the hepatopancreas. The result is the same as the findings in other crustaceans, such as* H. americanus* [17],* Fenneropenaeus chinensis* [36], and* E. sinensis* [15], and consistent with the report that hemocyanin synthesis occurs mainly in the hepatopancreas [37]. MnHc-1 expression was detected in all the examined tissues except for muscle, which is different from another subunit of hemocyanin in* M. nipponense* (MnHc-2) expressed in the muscle [26]. We also found that MnHc-1 was expressed in ovary, whereas MnHc-2 was hardly expressed in ovary [26]. The discrepant expression patterns of these two subunits of hemocyanin may be owing to their functional specialization in different tissues.

Hemocyanin is an important multifunctional protein in mollusks and arthropods. Besides its role as an oxygen carrier, its immune functions including antibacterial activities, agglutination property, and PO activity have become hot topics of immunological research [2, 4, 5, 7]. Zhang et al. [38] found that the main protein directly bound to the* Vibrio alginolyticus*,* Vibrio harveyi*,* A. hydrophila*, and* Staphylococcus aureus* in* L. vannamei* serum was hemocyanin, suggesting that hemocyanin possesses antibacterial functions. The C-terminus of* L. vannamei* hemocyanin is possibly related to the immunity in shrimp to different pathogens [39]. Gram-negative bacteria* A. hydrophila* are a common species of the* Aeromonas* genus in water and water habitants [40]. The infection of* A. hydrophila* in fish and prawn including* M. nipponense* has been one of the major diseases under farming conditions [25, 41]. Sun et al. [15] showed that the hemocyanin gene expression of* E. sinensis* was significantly upregulated by* A. hydrophila* infection. In our study, temporal and spatial expressions of MnHc-1 in the hemocytes of prawn infected with* A. hydrophila* showed a clear time-dependent pattern. The level of MnHc-1 mRNA expression significantly decreased at 3 h after injection, then started to significantly increase after 6 h and 12 h, and then reached the peak at 12 h, implying that the hemocyanin is involved in the antibacterial defense of prawn. In another study, the level of MnHc-2 mRNA expression in prawn significantly increased over time and peaked at 3 h after the* A. hydrophila* challenge [26]. Clearly, these two hemocyanin subunits respond quite differently in defense against bacterial infection. Transcriptional upregulation in MnHc-1 was found after 6 h of* A. hydrophila* injection. Lei et al. [4] found a similar result in* P. japonicus* that* PjHcL* transcriptional upregulation occurred after 4 h of injection of the active WSSV. It is possible that* PjHcL* may be triggered by the fast expressed proteins in virus. It is also likely that MnHc-1 may be induced by fast expressed protein in bacteria, but the detailed defensing mechanism of hemocyanin against bacterial infection needs further study.

The expression of hemocyanin subunits varies with environmental or nutritional changes [42, 43]. Copper is the metal in the center of a hemocyanin molecule [42]. In* E. sinensis*, expression of hemocyanin mRNA was affected by the level of dietary copper [15]. Our present study showed that the level of dietary copper affected the hemocyanin gene expression in prawn. The level of MnHc-1 mRNA in hepatopancreas of the prawn fed 43.1–157.1 mg Cu kg^−1^ diet was significantly higher compared to that fed 2.8–29.8 mg Cu kg^−1^. The increase of dietary Cu concentration also increased the Cu content in hepatopancreas, especially in the 43.1–157.1 mg Cu kg^−1^ groups. Hemocyanin synthesis mainly occurs in the hepatopancreas [37]. Therefore, we suggest that the high level of copper content in the hepatopancreas can trigger MnHc-1 mRNA expression, whereas the optimal level of dietary copper (20–40 mg kg^−1^ diet) can increase hemocyanin mRNA expression in the hepatopancreas and hemocytes of crab [15]. These studies suggest that the effect of dietary copper level on hemocyanin mRNA expression is species-specific. It seems that the two subunits of hemocyanin may have structural functions in their own hemocyanin and show different response to the level of dietary copper. Hemocyanin is a copper-containing protein that mainly carries oxygen in crustaceans [9]. Dietary copper may be first used for hemocyanin synthesis as low dietary copper did not reduce the level of MnHc-1 mRNA.

In conclusion, we cloned the hemocyanin subunit gene (MnHc-1) from the hepatopancreas of* M. nipponense*. Our results suggest that MnHc-1 may play a critical role in antibacterial defense in prawn. Accumulation of high copper levels in hepatopancreas of prawn triggers MnHc-1 gene expression. These results provide the foundation for further studies in biological function and regulation of hemocyanin in crustacean.

## Figures and Tables

**Table 1:** Ingredients and compositions of experimental diets (%).

Ingredient	Percentage of dry weight
Casein^a^	30
Fish meal^b^	20
Corn starch	26
Fish oil^c^	4
Soybean oil^d^	2
Vitamin mix^e^	2
Cu-free mineral mix^f^	3
Attractant^g^	3
Cholesterol^h^	0.5
Choline chloride^h^	0.5
Lecithin^h^	0.5
Cellulose^h^	6.5
Sodium carboxymethylcellulose^h^	2
Proximate composition	
Crude protein	40.6
Crude lipid	7.47
Crude ash	7.02

^a^Sigma-Aldrich Co., Shanghai, China.

^b^Tecnologica De Alimentos USA.

^c^Xiamen Xinsha Pharmaceutical Co. Ltd., Xiamen, China.

^d^National Golden Dragon Fish Co. Ltd., Shanghai, China.

^e^Vitamin mixture (mg/100 g mixture): vitamin A 420000 IU; vitamin C 6000 mg; *α*-tocopherol acetate 2000 mg; vitamin D3 120000 IU; vitamin K 1000 mg; vitamin B1 1000 mg; vitamin B2 1000 mg; vitamin B6 1600 mg; vitamin B12 2 mg; niacin 5000 mg; folic acid 400 mg; inositol 6000 mg; biotin 10 mg; and calcium pantothenic 3500 mg, Hangzhou Minsheng Bio-Tech Co., Ltd., China.

^f^Composition of mineral mixture (g/kg diet): KCl 0.84, MgSO_4_·7H_2_O 3, NaH_2_PO_4_ 6.45, KH_2_PO_4_ 3, Ca(H_2_PO_4_)_2_·H_2_O 7.95, CaCO_3_ 3.15, C_6_H_10_CaO_6_·5H_2_O 4.95, FeC_6_H_5_O_7_·5H_2_O 0.36, ZnSO_4_·7H_2_O 0.1428, MnSO_4_·H_2_O 0.0321, Na_2_SeO_3_ 0.0009, AlCl_3_·6H_2_O 0.0045, CoCl_2_·6H_2_O 0.042, and KI 0.0069.

^g^Alanine 0.6%, glycine 0.6%, glutamic acid 0.6%, and betaine 1.2%.

^h^China National Medicine Corporation Co., Ltd., Beijing, China.

**Table 2:** Primers used in our study.

Name	Sequence (5′-3′)
MnHc-s1	GTCGACTCTACTCCTCTTGG
MnHc-a1	TCGGTTATCCTTCAGCTC
MnHc-s2	TTCTGCTGATGCCTCCAA
MnHc-a2	TTCTTCACGGTGCCTGTC
MnHc-5′GSP	GGCGGTGAACAGCTTCTCCATTATC
MnHc-3′GSP	GCATGATGTGAACTTCCTCCTGTGG
5′-RACE CDS primer A	(T)_25_V N (N = A, C, G or T; V = A, G or C)
3′-RACE CDS primer A	AAGCAGTGGTATCAACGCAGAGTAC(T)_30_ V N
UPM	CTAATACGACTCACTATAGGGCAAGCAGTGGTATCAACGCAGAGT
	CTAATACGACTCACTATAGGGC
*β*-Actin-s	GTGCCCATCTACGAGGGTTA
*β*-Actin-a	CGTCAGGGAGCTCGTAAGAC
